# Mesoporous Silica Nanoparticles for Co-Delivery of Drugs and Nucleic Acids in Oncology: A Review

**DOI:** 10.3390/pharmaceutics12060526

**Published:** 2020-06-08

**Authors:** Juan L. Paris, María Vallet-Regí

**Affiliations:** 1 Allergy Research Group, Instituto de Investigación Biomédica de Málaga-IBIMA, Hospital Civil, 29009 Málaga, Spain; 2 Nanostructures for Diagnosing and Treatment of Allergic Diseases Laboratory, Andalusian Center for Nanomedicine and Biotechnology-BIONAND, 29590 Málaga, Spain; 3 Departamento de Química en Ciencias Farmacéuticas (Unidad Docente de Química Inorgánica y Bioinorgánica), Facultad de Farmacia, Universidad Complutense de Madrid, Instituto de Investigación Sanitaria Hospital 12 de Octubre (imas12), 28040 Madrid, Spain; 4 Centro de Investigación Biomédicaen Red de Bioingeniería, Biomateriales y Nanomedicina (CIBER-BBN), Spain

**Keywords:** mesoporous silica nanoparticles, co-delivery, drug delivery, nucleic acids, gene transfection, nanomedicine

## Abstract

Mesoporous silica nanoparticles have attracted much attention in recent years as drug and gene delivery systems for biomedical applications. Among their most beneficial features for biomedicine, we can highlight their biocompatibility and their outstanding textural properties, which provide a great loading capacity for many types of cargos. In the context of cancer nanomedicine, combination therapy and gene transfection/silencing have recently been highlighted as two of its most promising fields. In this review, we aim to provide an overview of the different small molecule drug-nucleic acid co-delivery combinations that have been developed using mesoporous silica nanoparticles as carriers. By carefully selecting the chemotherapeutic drug and nucleic acid cargos to be co-delivered by mesoporous silica nanoparticles, different therapeutic goals can be achieved by overcoming resistance mechanisms, combining different cytotoxic mechanisms, or providing an additional antiangiogenic effect. The examples here presented highlight the great promise of this type of strategies for the development of future therapeutics.

## 1. Introduction

The use of nanoparticles for cancer therapy has gathered great attention from the scientific community [1,2]. This interest was initially associated with the observation that macromolecules and particles in the nanometer range could accumulate in tumor tissues [3], a general trend that could be further improved by decorating the nanoparticle surface with active targeting molecules [4,5]. Even though the mechanism through which nanoparticles accumulate into tumors has been recently put into question [6,7], several nanoformulations have reached the clinical market, thanks to improved performance over alternative formulations [8,9,10]. In the context of the most recent clinically approved nanoformulations, two areas of nanomedicine appear as especially promising for cancer therapy: nucleic acid (or “gene”) delivery [11] and combination therapy [12].

Nanotechnology-based nucleic acid delivery can be highlighted by the recent approval of the first nanoparticle-based gene delivery system (Onpattro^®^ [13]). The approval of Onpattro^®^, a lipid-based RNA-delivery system for the treatment of hereditary ATTR amyloidosis, opens the door to countless opportunities in the field of nucleic acid delivery, for a plethora of different therapeutic applications. By delivering different nucleic acid therapeutics to specific cells, the expression of certain proteins can be induced or inhibited (silenced) [14]. Since the vast majority of therapeutically-useful nucleic acids cannot cross the cell membrane of target cells without first being destroyed by DNAses or RNAses in the biological environment, achieving intracellular delivery is a key factor to ensure therapeutic efficacy [11]. However, the specific subcellular location that has to be reached changes with the type of nucleic acid cargo. For example, plasmid DNA needs to reach the cell nucleus in order to be expressed and give rise to the formation of the desire protein [15]. On the other hand, interfering RNA (such as short interfering RNA or siRNA, constituted by double-stranded RNA of 20–25 base pairs in length) inhibits the expression of certain messenger RNA (mRNA) when delivered to the cell cytoplasm [15]. Therefore, the characteristics of the carrier nanoparticle have to be tailored to the type of nucleic acid being delivered and the desired application, to ensure successful delivery of the cargo to the target cells and in the appropriate subcellular location. In this regard, and since most nanoparticles enter inside cells by endocytosis, escaping the initial location in the endosomal compartment is key to ensure their correct performance.

The importance of nanomedicine in combination therapy for cancer can be showcased by the recent approval of the first nanoparticle-based drug combination co-delivery system, Vyxeos^®^ [16]. Vyxeos^®^ is a daunorubicin/cytarabine combined liposomal formulation that achieved extended overall survival in acute myeloid leukemia patients [16]. The strategy of joining nucleic acid delivery with combination therapy in a single nanoparticle vehicle appears, therefore, to be extremely promising for the development of future therapies. Even though many different types of nanoparticles have been proposed for the co-delivery of drugs and nucleic acids, mesoporous silica nanoparticles (MSNs) seem particularly promising thanks to their outstanding properties, which have turned MSNs into one of the most widely employed systems in nanomedicine research in recent years [17,18]. While many different review articles have addressed MSN development for therapeutics, we think that an update of recently reported work in their use for co-delivery of small molecule drug and nucleic acids is lacking. The objective of this work is therefore to provide an overview of the co-delivery strategies reported using MSNs as the carrier system. To do this, the main characteristics of MSNs for drug delivery are summarized first, followed by a section classifying the nanoparticle design options for nucleic acid delivery with MSNs.

## 2. Mesoporous Silica Nanoparticles for Drug Delivery

**Figure 1:**
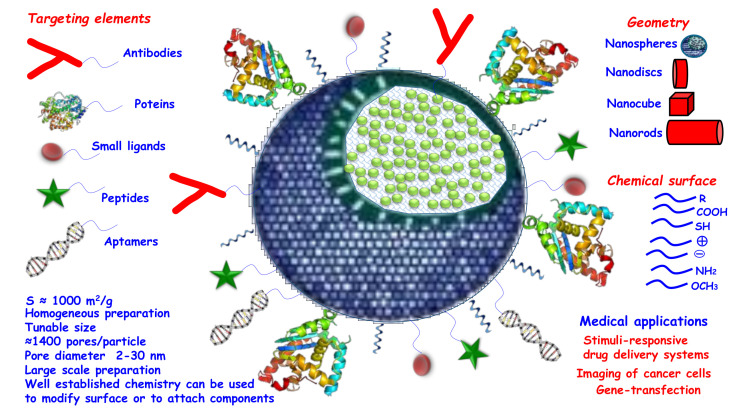
Schematic representation of characteristics of mesoporous silica nanoparticles (MSNs) for biomedical application.

Mesoporous materials (with pores between 2 and 50 nm [19]) were developed in the early 1990s and were used for multiple applications, such as catalysis or drug delivery [20,21,22]. Mesoporous silica materials consist of an amorphous silicon dioxide (silica) matrix with ordered porosity in the mesoporous range. Among the main characteristics that make mesoporous silica materials an attractive option for drug delivery systems are their textural properties, their physical-chemical stability and their ease of chemical modification [23]. In terms of their textural properties, they have a high surface area, up to 1500 m^2^/g, and a high pore volume, around 1 cm^3^/g, which allows them to hold large quantities of drugs inside [24]. Their easy synthesis and the established silanol chemistry enable many different physical and chemical modifications of MSNs to tune the particles for specific applications (Figure 1). For example, by modifying the chemistry of the pore surface and their size, MSNs can be tailored for efficiently loading a wide variety of different drugs, both hydrophobic and hydrophilic [25,26,27,28,29,30].

Regarding toxicological aspects, MSNs are generally considered to be biocompatible and well tolerated [31,32]. Although some inconsistencies have been found between in vitro and in vivo results, the main types of tissue damage caused by silica particles tend to correlate with thrombosis derived from particle aggregation [32]. It is worth noting that the aggregation potential of silica nanoparticles can be greatly decreased by modifying the nanoparticle surface, for example by grafting poly(ethylene glycol) (a process known as PEGylation) [33]. Besides this, MSNs are known to degrade in the biological environment into non-toxic products that can be safely excreted [34]. As long as the silica doses are not too large, this would prevent chronic accumulation in the organism. A recent study that evaluated the one-year toxicological behavior of intravenously-injected single-dose silica nanoparticles in mice has shown that, while large (500 nm) non-porous silica particles produced some lung, heart and kidney damage attributed to resolved thrombosis, no significant toxicity was produced by MSNs and smaller non-porous silica particles [35].

One of the most explored areas of research in the context of MSNs for drug delivery is the development of intelligent mesoporous materials that are capable of inducing the release of their load in the presence of certain stimuli [36]. Due to the open porous structure of the mesoporous silica materials, with the aim of controlling the release from them, it becomes necessary to anchor a second component on the pore openings, which acts as a gate capable of regulating the release of the content present in the mesopores. The nature of the gates that can be anchored to MSN is very broad, from polymers [37] to smaller compact nanoparticles [38]. The connector through which the gate is anchored, or some of the components of the gate itself are designed to produce a response (change of shape, breakage, or others) in the presence of the stimulus of interest [39]. Through this type of strategy, MSNs have been developed that are sensitive to a multitude of stimuli, both internal (pH [40], redox [41], enzymes [42]) and external (light [43], magnetic field [44,45], ultrasound [46,47]).

MSN drug carriers have been developed for a wide variety of therapeutic applications, such as anesthesia [48], infection [49,50], osteoporosis [51] and allergy [52], among others. However, the most studied application by far is cancer therapy [53], developing MSNs to deliver anticancer drugs such as doxorubicin [37,54,55,56,57,58], camptothecin [59], topotecan [60], paclitaxel [61], gemcitabine [62], and 5-fluorouracil [63].

## 3. Mesoporous Silica Nanoparticles for Gene Delivery

**Figure 2:**
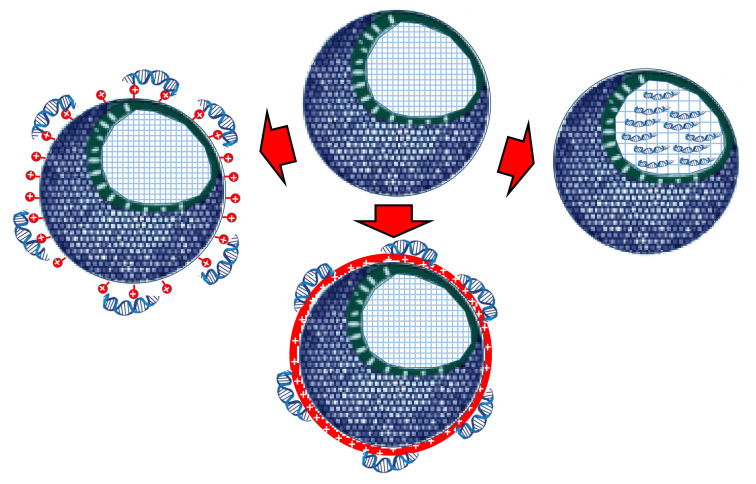
Schematic representation of different strategies to carry nucleic acids in MSNs: Nucleic acid delivery by surface-functionalized MSNs (**left**), nucleic acid delivery by polycation-coated MSNs (**center**) and nucleic acid delivery within MSN pores (**right**).

Regarding the use of silica particles for nucleic acid delivery, non-porous silica nanoparticles were initially explored. By modifying silica particles with positively-charged organic moieties, intracellular nucleic acid delivery becomes possible, at least in vitro [64,65]. Not only can surface-modified non-porous silica particles be used for gene transfection, but Miyata et al. have also shown that polyplexes (complexes of cationic polymers with nucleic acids) can be coated with silica to improve their performance as gene delivery agents [66]. Other works have reviewed many of the proposed strategies for gene delivery using non-porous silica nanoparticles [67]. More interestingly, and taking advantage of the properties already mentioned in the previous section, MSNs have been employed to carry nucleic acids into different types of cells through different strategies [68,69] (Figure 2). First, the surface of the MSNs can be modified to present positively charged moieties that can interact with negatively charged nucleic acids, enabling their transport into cells. The second option, which has become the most common strategy for gene transfection with MSNs, consists in coating MSNs with a polycationic component, which the negatively charged nucleic acid cargo will be inserted in for transport. In this strategy, the polycation can be adsorbed on the MSN surface by electrostatic interactions or it can be covalently grafted, which will modify the behavior of the composite system. Finally, a more recent approach has consisted in preparing novel MSNs with enlarged pores in which the cargo nucleic acid can be loaded. This leaves the nanoparticle surface available for different chemical modifications aimed at providing different capabilities, such as specific cell targeting or prolonged circulation, among others.

### 3.1. Nucleic Acid Delivery by Surface-Functionalized MSNs

The most common strategy for nucleic acid delivery by surface-functionalized MSNs is to decorate the nanoparticle surface with positively charged amino groups (for example, using (3-Aminopropyl) triethoxysilane or APTES). These amino groups on the nanoparticle surface will interact with negatively charged phosphate groups of nucleic acids, enabling them to carry the nucleic acid cargo inside cells. The following examples have been selected to illustrate the variety of nucleic acid cargos that can be used with this strategy for different biomedical applications.

Surface amino-functionalized MSNs were seen to provide in vivo transfection of a platelet derived growth factor B (PDGFB) plasmid in the context of Achilles tendon healing, even though the transfection efficiency of the same particles in vitro appeared to be negligible [70]. Chen et al. also prepared positively-charged MSNs that were shown to deliver the hepatocyte nuclear factor 3β (HNF3β) plasmid to induced pluripotent stem cells (iPSCs), inducing differentiation into hepatocyte-like cells [71]. Similarly, Kim et al. used amine functionalized MSNs to complex a bone morphogenetic protein-2 (BMP2) plasmid to transfect mesenchymal stem cells (MSCs), successfully inducing their osteogenic differentiation [72]. Chang et al. also used amino-functionalized MSNs to deliver genetic material to iPSCs in order to induce differentiation, in this case towards dopaminergic neurons [73]. However, in this case, the authors had to co-deliver two types of nucleic acids to achieve successful differentiation: Nurr1 plasmid and Rex1 siRNA. With this combined DNA/siRNA delivery system, over 88% of the treated cells expressed dopamine transporter after transfection by MSNs.

Other modifications besides amine functionalization can also be performed to provide other capacities to the nanosystem. For example, Brevet et al. prepared amino- and histidine-functionalized MSNs that improved their in vivo transfection capacity with a therapeutic plasmid by enhancing endosomal escape, thanks to the histidine moiety [74]. When evaluating different MSN surface functionalization possibilities for plasmid delivery, Mahmoodi et al. observed a better performance of imidazolium-modified MSNs when compared with amino-functionalized ones [75].

In a different therapeutic approach, Tao et al. prepared amino-functionalized MSNs to deliver immunostimulatory double stranded DNA (dsDNA) into Raw 264.7 cells [76]. With this system, an improved behavior with larger in vitro interferon-α production was achieved, compared to the standard procedure using the (1,2-dioleoyl-3-trimethylammonium-propane) (DOTAP) lipid as carrier. This result highlights the great potential of using functionalized MSNs as delivery agents of immuno-stimulatory DNA drugs.

### 3.2. Nucleic Acid Delivery by Polycation-Coated MSNs

Similar to the previous section, the surface of MSNs is also modified to provide a positive charge to enable nucleic acid transport. However, by using polycationic coatings in which the nucleic acid is inserted, this not only enables intracellular delivery of the nucleic acid, but this might also protect the cargo from extracellular degradation, by hindering its interaction with DNAses and RNAses present in the biological environment. The main types of polycations used for this are dendrimers and polyethyleneimine (PEI), although a variety of other polymers have also been studied, as can be seen in the examples below.

Radu et al. obtained MSNs modified with G2 Polyamidoamine Dendrimers as capping agents, which were also used for cell transfection with a GFP plasmid [77]. MSNs with second or third generation carbosilanedendrons grafted on their surface were also later shown to enable successful internalization of the vector carrying single stranded DNA without affecting the cellular viability [78].

Yiu et al. developed magnetic core-containing mesoporous silica particles coated with PEI for the in vitro delivery of plasmidic DNA [79]. A targeting agent can also be included in this type of nanoparticle design, to provide some specificity in the target cell population where uptake and transfection will take place. For example, MSNs with a mannosylated PEI coating were obtained to deliver DNA to macrophages [80]. Later work has shown that PEI-coated MSNs can be used not only to deliver plamidic DNA, but also other types of nucleic acids, such as siRNA [61,81]. Furthermore, the positive surface charge provided by these cationic polymeric coatings also enhances nanoparticle uptake [61]. As an example for the delivery of a therapeutic siRNA, Ngamcherdtrakul et al. demonstrated the effective delivery of a siRNA against the human epidermal growth factor receptor type 2 (HER2), by a PEI-coated MSN system [82]. Even though most of the mentioned works relied on electrostatic interactions between the silica surface and PEI, He at al. showed that when PEI was covalently grafted on the surface of MSNs, the obtained particles were resistant to serum, presenting better transfection capacity than PEI/DNA polyplexes, and with reduced cytotoxicity [83]. The PEI-coated MSN system has become so widespread among the scientific community that some studies have already been performed trying to evaluate different processing options, to enable long-term storage that ensure that the hybrid system maintains all of its properties and biological behavior. For example, Zhang et al. reported that by incorporating the cryoprotectanttrehalose, PEI-coated MSNs could be lyophilized, maintaining their integrity for at least 4 months at room temperature, which would enable the mass production and distribution of this system for therapeutic application [84]. 

Other polycations have also be employed to enable nucleic acid delivery when coating MSNs, such as poly(2-(dimethylamino)ethylmethacrylate) (PDMAEMA) and poly (2-(diethylamino)ethylmethacrylate) (PDEAEMA) [85]. With these systems, Bhattarai et al. not only showed the feasibility of employing other polycations for DNA and siRNA delivery, but they also showed that loading these composite particles with chloroquine enhanced their transfection capacity. Chloroquine acted here by breaking the endo-lysosomes in which the particles are trapped after endocytosis, releasing them into the cytoplasm and enhancing the delivery of the cargo nucleic acid to its intracellular target (nucleus for DNA or cytoplasm for siRNA). However, when this strategy was tested in a different MSN-based system with PEI as coating polymer, Zarei et al. observed that the introduction of chloroquine increased the cytotoxic effect of the particles without improving their transfection efficiency [86]. Lin et al. later developed MSNs with PDMAEMA covalently grafted through disulfide bonds. When the particles were incubated with cells while carrying a siRNA sequence inserted in the polymeric component, the reducing intracellular environment led to the cleavage of the disulfide bonds, detaching the polymer and easing siRNA release [87]. Through this mechanism, the particles showed an improved gene silencing capacity, even when compared with the standard Lipofectamine 2000. Finally, this system was shown to deliver a therapeutic siRNA (siPlk1) to a tumor, producing tumor suppression in a xenograft mouse model. The effect of MSN particle size and morphology in the toxicity and transfection efficiency of these PDMAEMA-coated systems has also been studied, showing that 300 nm long chiral nanorods acted as the most efficient gene carriers, compared to 100 nm spheres, 200 nm chiral rods and 300 nm non-chiral rods [88]. 

More recently, Kar et al. prepared poly-l-arginine-grafted MSNs which were capable of transfecting HeLa cells with a plasmid encoding for the fluorescent protein mCherry [57]. Interestingly, in that work, it was observed that poly-l-arginine-grafted MSNs were much more efficient at transfecting HeLa cells than analogous poly-L-arginine-grafted non-porous silica nanoparticles. The authors hypothesized that this difference was caused by a higher density of poly-l-arginine on the surface of non-porous silica particles, which prevented the plasmid from being released in an efficient way.

### 3.3. Nucleic Acid Delivery within MSN Pores

**Figure 3:**
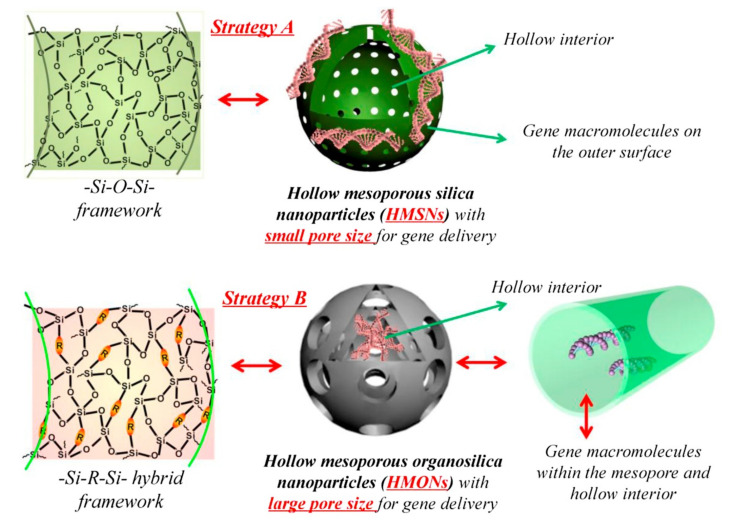
Schematic representation of the possible strategies for nucleic acid delivery using hollow MSNs: particles with small pore sizes can only carry the nucleic acid on their surface (top), while particles with large pores can carry the nucleic acid cargo within them. This image is used without modifications from [74]. Copyright© 2016, Elsevier.

MSNs can also act as gene carriers by introducing the DNA cargo within its mesopores. However, most of the mesoporous materials employed for drug delivery present pores that are too small to carry therapeutically interesting DNA molecules. Therefore, nucleic acids can only be carried on the nanoparticle surface unless MSNs with large enough pores are obtained (Figure 3). For this reason, Gao et al. prepared MSNs with large pore sizes (around 20 nm) that enable the loading and release of plasmids [89]. Zhu et al. later prepared large pore organically-functionalized and PEI-coated hollow MSNs that could also be used to deliver a hepatocyte growth factor (HGF) plasmid into bone marrow-derived MSCs in the context of regenerative medicine [90].

Besides loading DNA within mesopores, Li et al. prepared MSNs loaded with siRNA molecules within the mesopores, in addition to further siRNA cargo loaded within a PEI coating [91]. Kim et al. also prepared MSNs with ultra-large 23-nm amino-functionalized pores capable of efficiently deliver plasmid DNA into cells [92]. The same authors later employed the same particles to deliver a vascular endothelial growth factor (VEGF) siRNA in vivo, inhibiting tumor growth after intratumoral injection. Möller et al. developed another siRNA delivery system, based on novel stellate core–shell MSNs with medium-size pores capped with a modularly designed cationic polymer [93]. In this system, the authors were capable of optimizing the conditions to achieve maximal siRNA delivery through differentially modifying their internal and external surface chemistry. In order to provide siRNA-carrying MSNs with endosomal escape capabilities (since endosomal retention is one of the main bottlenecks in nucleic acid delivery), Li et al. grafted the fusogenic peptide KALA to the PEI coating of MSNs with siRNA loaded within their mesopores [94]. In this way, the particles provided successful gene silencing both in vitro and in vivo, even achieving tumor growth inhibition when delivering a siRNA targeting vascular endothelial growth factor (VEGF). This same enlarged-pore strategy can also be used to develop stimuli-responsive gene transfection agents. For example, Li et al. prepared MSNs with large conical pores functionalized with disulfide and amide bond-containing linkers with a positively-charged ammonium group at their end [95]. This assembly enables the loading of both plasmid DNA and siRNA, which would be released after cellular uptake as a consequence of the cleavage of the linkers, by either acidic endosomal pH or the reducing intracellular environment. As another example, Prabhakar et al. also developed a redox-responsive siRNA delivery system based on MSNs with expanded mesopores capped with hyperbranched PEI grafted through redox-cleavable disulfide bonds [96]. After cellular uptake, and thanks to the intracellular reducing environment, the system was capable of releasing a cell-killing siRNA in a sustained manner for several days, showing very promising in vitro biological activity. An alternative strategy to achieve prolonged siRNA delivery without using polymers as capping agents is coating the particles with lipid bilayers, a nanosystem known as protocells [97]. Ashley et al. prepared protocells for siRNA delivery based on large pore (23–30 nm) MSNs and coated by actively-targeted lipid bilayers [98]. These protocells had a 10- to 100-fold greater siRNA loading capacity than the equivalent liposomes without silica component. They were also shown to produce complete silencing of the expression of cyclin A2, B1, D1, and E.

Other types of therapeutic RNAs have also recently been introduced in MSNs: small hairpin RNA (shRNA) [99] and microRNA (miRNA) [100]. shRNAs are artificial RNA structures that also act as gene silencing agents. Zhang et al. prepared Large-Pore MSNs that could effectively deliver a shRNA directed towards the tumor necrosis factor receptor-associated factor-3 (shRNA-TRAF3) into Kupffer cells, both in vitro and in vivo [101]. miRNAs are non-coding RNAs which regulate the expression of various genes, many involved in cancer progression. Besides using miRNAs for diagnosing and evaluating prognosis in cancer patients, it has also been recently proposed to deliver miRNA mimics or anti-miRs for therapeutic use [100]. As examples of these strategies, Li et al. have prepared MSN-based systems capable of delivering anti-mir-155 or miR-328, both in the context of colorectal cancer therapy [102,103]. Similar approaches have been developed for other cancer types, such as triple negative breast cancer (TNBC), where miR-34a is down-regulated and miR-10b is up-regulated. By co-delivering miR-34a-mimic and antisense-miR-10b in MSNs, Ahir et al. achieved the efficient inhibition of tumor growth and the retardation of metastasis in an in vivo model of TNBC [104].

## 4. Mesoporous Silica Nanoparticles for Co-Delivery of Nucleic Acids and Small-Molecule Drugs

The most common strategies discussed in the previous section consist of employing surface functionalizations or a polycationic coating on the surface of MSNs to complex nucleic acids and carry them within target cells. This leaves the inner mesopore volume available to carry different drugs which can be carefully selected to provide synergistic effects when combined with the one provided by the nucleic acid being co-delivered [105,106]. A proof-of-concept of this kind of strategy can be seen in the work of Zhu et al., who developed an enzyme-responsive poly(l-lysine) (PLL)-coated hollow MSN system, that could co-release fluorescein and a cytosine-phosphodiester-guanine-oligodeoxynucleotide, as models for small molecule drugs and nucleic acids, respectively [69]. Another proof-of-concept work, in this case by developing a near infrared light-responsive system, was developed by Chang et al. [107]. With mesoporous silica-coated gold nanorods, the authors successfully prepared a system that could co-deliver Dox and dsDNA or a GFP siRNA. As a recent example for non-cancer application, PEI-coated MSNs have also been developed to co-deliver the therapeutic peptide osteostatin and a SOST siRNA for osteoporosis treatment [51]. By co-delivering a therapeutic agent that stimulates bone production (osteostatin) and another one that inhibits bone resorption (SOST siRNA), a synergistic effect in increasing osteogenic markers was achieved. Another MSN-based system co-delivering curcumin and RhoG-DsRed plasmid was more recently prepared by Cheng et al. for promoting neurite outgrowth [108].

The following examples have been selected to illustrate the different therapeutic strategies that can be developed by co-delivering small molecule drugs and therapeutic nucleic acids with MSNs for oncology (Table 1). The different drug/nucleic acid combos studied can be classified according to the therapeutic aim that the nucleic acid exerts in the combination. In this way, the selected nucleic acid can prevent resistance towards the chemotherapeutic drug by non-pump or pump-mediated mechanisms; it can be involved in inducing cancer cell death by a different mechanism than the small molecule drug, either directly or by activating a prodrug, and it can provide anticancer therapeutic efficacy through the inhibition of angiogenesis.

### 4.1. Avoiding Non-Pump Resistance

Chen et al. used MSNs coated with G2 polyamidoamine dendrimers to co-deliver the drug doxorubicin (Dox) and a Bcl-2 siRNA [109]. In this work, Dox would be responsible for inducing cancer cell death by inducing apoptosis, while the used Bcl-2 siRNA would act as a suppressor of cellular antiapoptotic defense (the Bcl-2 protein is the main player for non-pump cancer chemotherapy resistance). This would enable efficient cancer therapy in some multidrug-resistant cancer cells. Ma et al. developed a redox-responsive system based on the same dual cargo system Bcl-2 siRNA/Dox, showing significant gene silencing in addition to the cytotoxic effect of Dox in an in vivo zebrafish model [110]. In this system, a cyclodextrin-based positively charged capping system was grafted through a disulfide bond on the MSN surface. This capping agent achieved two different objectives: first, it prevented the premature release of the small molecule cargo (Dox), which was loaded within the mesopores. Second, its positive charge enabled it to interact with the cargo siRNA. When the nanosystem gets to the intracellular environment, the disulfide bonds are reduced, releasing both Dox and the siRNA. Another reducible co-delivery system for Bcl-2 siRNA and Dox was also prepared by Zhao et al., but in this case, the disulfide linker was used to graft the siRNA directly to the nanoparticle surface, simultaneously acting as the capping agent for the mesopore-loaded Dox [111]. This system also showed improved therapeutic efficacy in an in vivo human breast adenocarcinoma MCF-7 xenograft mouse model. PEGylated PEI-coated MSNs have also been developed to co-deliver Bcl-2 siRNA, with epirubicin as another chemotherapeutic drug, enhancing the antitumor effect both in vitro and in vivo, compared to particles carrying only the drug [112].

**Figure 4:**
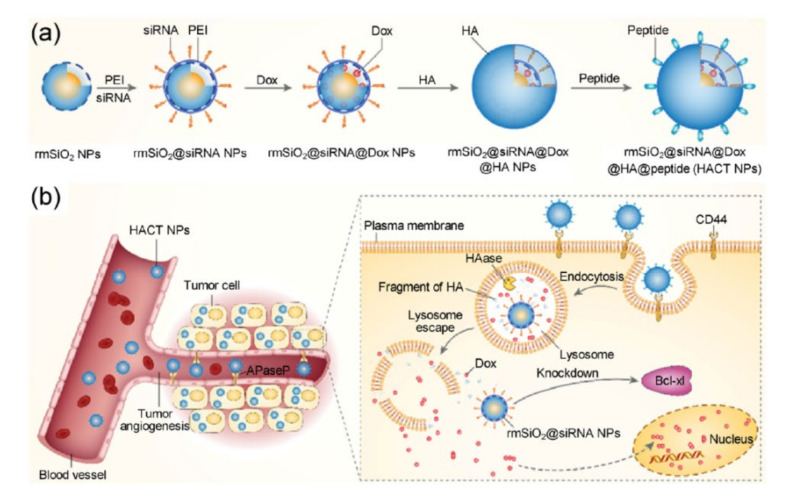
(**a**) Schematic representation of multilayered hyaluronic acid- polyethyleneimine (PEI)-rattle MSNs, with a cascade of two targeting agents (hyaluronic acid, HA, and peptide) and two cancer therapeutic agents (connective tissue growth factor (CTGF) siRNA and Dox). (**b**) Schematic representation of the therapeutic mechanism of CTGF siRNA and Dox co-loaded multilayered hyaluronic acid-PEI-rattle MSNs for the treatment of CTGF overexpressing breast cancer. This image is used without modifications from [99]. Copyright© 2017, Springer.

Other proteins involved in non-pump chemotherapy resistance are survivin and connective tissue growth factor (CTGF). Survivin is an inhibitor of apoptosis which is involved in controlling cell proliferation. Li et al. prepared pH-responsive MSN particles for the co-delivery of Dox and a survivin shRNA-Expressing Plasmid, which decreased the proliferation of cancer cells in vitro, as well as inhibited tumor growth in vivo [113]. On the other hand, CTGF overexpression is known to induce the upregulation of Bcl-xL and the cellular inhibitor of apoptosis protein 1 (cIAP1). multilayered hyaluronic acid-PEI-rattle MSNs prepared to co-deliver CTGF siRNA and Dox produced improved in vivo anti-cancer efficacy, compared with only chemotherapy, in an MDA-MB-231 tumor bearing mice model (Figure 4) [114].

### 4.2. Avoiding Pump-Mediated Resistance

Another main mechanism driving chemotherapy resistance in tumors is pump-mediated resistance. In the most common type of pump-mediated resistance, cancer cells acquire the ability to produce different types of pumps capable of expelling chemotherapeutic drugs to the extracellular environment, greatly increasing the necessary concentration of drugs to achieve therapeutic effect. Among the different types of pumps known to exert this function, P-glycoprotein (Pgp, also known as multiple drug resistance protein, MDR) is probably the most well-known. Menget al. developed PEI-coated MSNs to co-deliver Dox and a siRNA inhibiting the production of Pgp [115]. With this dual delivery system, the authors observed an increase in the cytotoxic effect of the particles incubated with a multidrug resistant cell line when compared to nanoparticles delivering just the drug. The same authors later improved the system by delivering an optimized ratio of Pgp siRNA and Dox with PEGylated PEI-coated MSNs in an in vivo model of multidrug-resistant breast cancer, achieving synergistic inhibition of tumor growth with significant Pgp knockdown at heterogeneous tumor sites [116]. A very similar system was used by Wang et al. in the context of squamous carcinoma treatment [117]. In this work, PEI-coated MSNs were also used to co-deliver Dox and siRNA against MDR1 (Pgp1), achieving a huge decrease in tumor size in vivo (over 80% decrease after 28 days). A different type of pump-mediated resistance does not involve the direct pumping of the chemotherapeutic drug outside of the cancer cell, but is related to the overexpression of transmembrane Ca^2+^ channels [118]. This overexpression is associated with an increase of Ca^2+^ influx, which is involved in proliferative pathways. Wang et al. developed amino-functionalized hollow MSNs for co-delivery of T-type Ca^2+^ channel siRNA and Dox [118]. The obtained in vitro and in vivo results showed a good biocompatibility profile of the nanosystem, and a high therapeutic efficacy, even in a drug-resistant breast cancer model. siRNAs against pump- and non-pump-mediated resistance mechanisms can also be combined in a single nanocarrier in addition to different small molecule drugs, such as in the work of Taratula et al. [119] In that study, an MSN carrier co-delivering small molecule drugs (Dox and cisplatin) and siRNAs (against Bcl-2 and pump MRP1) was developed for inhale-based administration in the context of lung cancer.

### 4.3. Inducing Direct Cancer Cell Death

A third strategy that can be adopted when co-delivering nucleic acids and small molecule drugs in the context of cancer therapy is combining a chemotherapeutic drug with a nucleic acid that can induce cell death by itself, aiming to achieve a synergistic effect by exploiting two different cytotoxicity mechanisms simultaneously. An example of this strategy can be found in the work of Lin et al. [120]. In this work, the authors developed a redox-responsive MSN system coated with a chitosan derivative, to co-deliver Dox and a plasmid encoding the p53 protein. p53 plays a critical role in the organism, by inducing apoptosis in damaged cells or cells in which their genetic material has been significantly compromised. The p53 gene is often mutated in different types of human tumors and delivering it to the target cancer cells has been proposed as a therapeutic strategy, with great promise for future translation [121]. This co-delivery system would, therefore, provide the combined effect of both therapeutic molecules being delivered. Furthermore, the chitosan derivative grafted on the nanoparticle surface was linked through disulfide bonds that could be cleaved under the reducing intracellular environment. Once the nanoparticles have been internalized by cancer cells, the release of both Dox and a p53 plasmid would take place [120]. This concept was validated in vitro with HeLa cells by the greatly enhanced cytotoxic effect of the system carrying both therapeutics (36% cell viability) compared to the system carrying only Dox (51% viability) or p53 plasmid (75% viability). Another PEI-coated hollow MSN formulation was developed to co-deliver p53 plasmid and a chemotherapeutic drug, in this case bortezomib for the treatment of non-small cell lung cancer (NSCLC) [122]. In this work, the enhanced efficacy of the co-delivery was demonstrated on p53-mutant NSCLC cells in vitro. We have recently reported a strategy that is conceptually similar, but selecting an anti-TWIST siRNA as the therapeutic nucleic acid cargo [123]. TWIST is a transcription factor that is linked to cancer in the context of angiogenesis, metastasis, cancer stem cell phenotype, and drug resistance [123]. For this reason, we co-delivered this siRNA in combination with the chemotherapeutic drug daunorubicin for ovarian cancer therapy. The delivery system employed was based on core-shell Fe_3_O_4_@MSNs coated by a functionalized PEI polymer. The cytotoxic effect of the system combining both therapeutic cargos was enhanced even further by activating the core magnetic particles with oscillating magnetic field.

### 4.4. Inducing Indirect Cancer Cell Death through Expressing Prodrug-Activating Enzymes

An indirect strategy that has also been explored for gene-therapy applied to oncology is the introduction of genes encoding for prodrug-activating enzymes, a strategy commonly referred to as suicide gene therapy [124]. In this strategy, the genes involved encode proteins that can activate a non-toxic prodrug into toxic species. The selected gene can be expressed either in cancer cells directly, or in vector cells that are then delivered to the tumor. When the prodrug is then systemically administered, the enzyme present in the area to treat will convert it into a toxic product that will kill the cell producing the enzyme, as well as other surrounding cells (which is known as the bystander effect) [125]. In one of our most recent works, we selected, as therapeutic cargo, a plasmid encoding the proteins cytosine deaminase (CD) and uracil phosphoribosyltransferase (UPRT) [126]. When the non-toxic pro-drug 5-fluorocytosine (5-FC) is administered, CD and UPRT combined convert it into 5-Fluorouridine monophosphate (5-FUMP), an irreversible inhibitor of thymidylate synthase, a very toxic molecule known to induce a strong bystander effect. This plasmid was introduced in ultrasound-responsive PEI-coated hybrid MSNs to transfect tumor-tropic placental stem cells. The particles were shown to maintain their capacity to load small molecules into their mesopores, and release their small molecule cargo upon ultrasound exposure. Regarding plasmid delivery, the particles achieved significant transfection of the vehicle stem cells, with both a green fluorescent protein plasmid and a therapeutic CU:UPRT plasmid. In an in vitro co-culture study, placental stem cells carrying the nanoparticles were shown to induce cancer cell death only when both nanoparticles and non-toxic prodrug 5-FC were present. This work opens the possibility of using nanoparticle-containing tumor-migrating stem cells for co-delivering a cytotoxic small molecule drug within and a prodrug-activating set of enzymes for combined cancer therapy.

### 4.5. Inhibiting Angiogenesis

**Figure 5:**
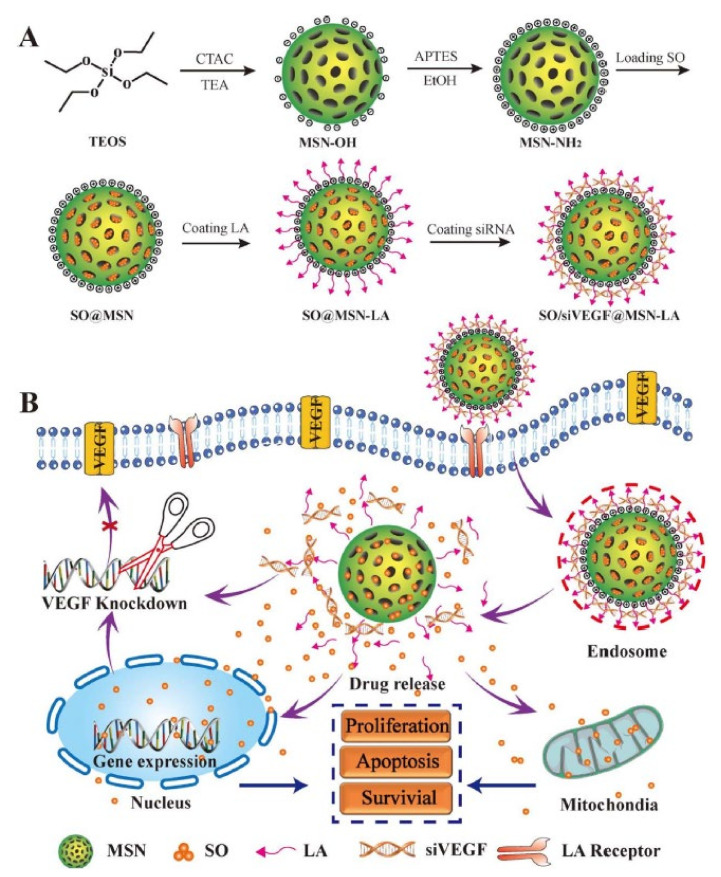
(**A**) Schematic representation of the preparation procedure of Sorafenib and vascular endothelial growth factor (VEGF) siRNA co-loaded modified MSNs; (**B**) Mechanism of the therapeutic effect. This image is used without modifications from [115]. Copyright© 2018, American Chemical Society.

An alternative to chemotherapeutic-based cancer therapy, in which a cytotoxic agent is directly employed to kill cancer cells, consists of preventing cancer cells from getting enough nutrients to survive. One way to achieve this is by attacking the tumor blood supply, preventing it from forming new blood vessels which are essential for its survival. This strategy is known as antiangiogenic therapy, and several types of drugs can be employed for this purpose, being one of the most common therapeutic targets, VEGF [127]. Delivering siRNA sequences directed against VEGF production is therefore an interesting approach with great potential, and it has already been mentioned in the previous section. However, a common limitation in antiangiogenic therapy is the appearance of the resistance mechanism against monotherapies [128], which is why combination therapies are usually employed, either with chemotherapeutic agents or with other anti-vascular therapeutics [129]. In this context, Zheng et al. developed a co-delivery nanosystem, based on MSNs for the treatment of hepatocellular carcinoma [130]. In this work, sorafenib (an antiangiogenic drug) was introduced inside MSN mesopores, while a VEGF siRNA was adsorbed on the nanoparticle surface by electrostatic interactions. This nanosystem, which was also actively targeted towards the asialoglycoprotein receptor by grafting lactobionic acid on the nanoparticle surface, was capable of inducing S cell cycle arrest and inhibiting the expression of VEGF in Huh7 cells in vitro (Figure 5).

**Table 1:** Small molecule and nucleic acid cargos co-delivered by MSN-based formulations for cancer therapy.

Small Molecule Cargo	Nucleic Acid Cargo	Responsive Release Trigger	In Vivo Model	Function of the Nucleic Acid	Reference
Doxorubicin	Bcl-2 siRNA	None	None	Avoiding non-pump resistance	[109]
Doxorubicin	Bcl-2 siRNA	Redox	Zebrafish	Avoiding non-pump resistance	[110]
Doxorubicin	Bcl-2 siRNA	Redox	Mouse	Avoiding non-pump resistance	[111]
Epirubicin	Bcl-2 siRNA	pH	Mouse	Avoiding non-pump resistance	[112]
Doxorubicin	Survivin shRNA-expressing plasmid	pH	Mouse	Avoiding non-pump resistance	[113]
Doxorubicin	CTGF siRNA	Hyaluronidase	Mouse	Avoiding non-pump resistance	[114]
Doxorubicin	Pgp siRNA	pH	None	Avoiding pump-mediated resistance	[115]
Doxorubicin	Pgp siRNA	pH	Mouse	Avoiding pump-mediated resistance	[116]
Doxorubicin	MDR1 (Pgp1) siRNA	None	Mouse	Avoiding pump-mediated resistance	[117]
Doxorubicin	T-type Ca^2+^ channel siRNA	pH	Mouse	Avoiding pump-mediated resistance	[118]
Doxorubicin and cisplatin	Bcl-2 siRNA and MRP1 siRNA	None	Mouse	Avoiding non-pump and pump-mediated resistance	[119]
Doxorubicin	p53 plasmid	Redox	None	Inducing direct cancer cell death	[120]
Bortezomib	p53 plasmid	pH	None	Inducing direct cancer cell death	[122]
Daunorubicin	Anti-TWIST siRNA	Oscillating Magnetic Fields	None	Inducing direct cancer cell death	[123]
Sorafenib	VEGF siRNA	pH	None	Inhibiting angiogenesis	[130]
Doxorubicin	VEGF siRNA	pH, Redox	Mouse	Inhibiting angiogenesis	[131]
Ursolic acid	VEGF siRNA	None	None	Inhibiting angiogenesis	[132]
Doxorubicin	VEGF shRNA	None	None	Inhibiting angiogenesis	[133]

This anti-angiogenic therapy strategy can also be combined with traditional chemotherapy agents, achieving improved therapeutic efficacy. In an example of this type of combination, Han et al. prepared multilayered nanoparticles with TAT-modified MSNs as a core [131]. Dox was included within the mesopores of the nanoparticles, before coating them with an intermediate anionic layer of poly(allylamine hydrochloride)-citraconic anhydride. These particles were then coated with an external cationic layer of galactose-modified trimethyl chitosan-cysteine, which was used to load a VEGF siRNA. The different components in the particle enabled uptake by target cells through actively targeted endocytosis, followed by endosomal escape and intracellular delivery of both Dox and the siRNA with active subcellular targeting to the cell nuclei (by the TAT peptide sequence). This multi-step nanosystem produced potent antitumor efficacies in vivo, even at low doses. In another example of this same type of combination, Zheng et al. developed a folic acid-targeted amino-functionalized MSN carrier that was loaded with ursolic acid within its mesopores and with VEGF siRNA through electrostatic interaction with the particle surface [132]. The promising in vitro results in this work demonstrate a great potential of this system, although no in vivo evaluation was reported at this point. Besides siRNA, other silencing nucleic acids have also been co-delivered with drugs for combined antiangiogenic- and chemotherapy. In this way, Li et al. prepared folic acid-targeted PEI-coated magnetic MSNs for the co-delivery of DOX and VEGF shRNA [133]. Even though this system was only evaluated in vitro by means of a human vascular endothelial cells (HUVEC)/HeLa co-culture model, the inclusion of a magnetic core adds an interesting possibility to further enhance therapeutic efficacy, by adding a third therapeutic modality through magnetic hyperthermia.

### 4.6. Future Directions

While many different MSN formulations have been developed to co-deliver small molecule drugs and nucleic acids in the context of cancer therapy, this strategy remains in the early stages of development, with the most advanced systems having only reached small animal testing. The proof-of-concept nature of most of these works implies that the formulations are likely not optimized for the best small molecule drug/nucleic acid ratio, and that the synthetic procedure is not easily scalable. This will impose many limitations when clinical translation is attempted, since a simple and cost-effective large-scale production of the formulation would be necessary. Therefore, ensuring a reproducible large-scale production scheme of the MSN carrier is of outmost importance. In parallel, further validation of the most promising formulations in more complex animal models will also be necessary. Many systems will not perform at the same level in more clinically relevant models as initially observed, which will force modifications of the nanosystems. On a broader nanomedicine context, the recent proposal that nanoparticle accumulation in solid tumors is driven by an active transcytosis process instead of a passive extravasation [7] also paves the way for a large set of new strategies for cancer nanotherapy. Finally, clinical trials would be necessary to ensure the safety and efficacy of these formulations. To the best of our knowledge, no clinical trial has been carried out on the systemic administration of MSNs. As a consequence, we believe that to clinically validate the good biocompatibility profile that MSNs have shown in animals, simpler formulations should probably be tested first, with a large impact of the desired application and administration route in the design of these trials. Despite all of the work that is still ahead of us to achieve clinical translation, the promising results available and the vast amount of knowledge gathered regarding these systems in recent years show remarkable potential of MSNs for combination cancer therapy.

## 5. Conclusions

Mesoporous silica nanoparticles constitute a promising agent for the development of drug and nucleic acid co-delivery systems. By carefully selecting both cargos to be loaded into targeted and stimuli-responsive systems, great therapeutic efficacy can be observed, at least in small animal models. The next step in this field of research will consist of selecting the most promising formulations and optimizing large scale production systems that will enable the validation of the proof-of-concept initial studies in more clinically-relevant models, and eventually, the development of clinical trials.
